# Rab6 Dependent Post-Golgi Trafficking of HSV1 Envelope Proteins to Sites of Virus Envelopment

**DOI:** 10.1111/tra.12134

**Published:** 2013-11-18

**Authors:** Helen L Johns, Claudia Gonzalez-Lopez, Charlotte L Sayers, Michael Hollinshead, Gillian Elliott

**Affiliations:** 1 Section of Virology, Faculty of Medicine, Imperial College LondonLondon, W2 1PG, UK; 2 Current address: Department of Microbial & Cellular Sciences, Faculty of Health & Medical Sciences, University of SurreyGuildford, GU2 7XH, UK

**Keywords:** *Gaussia* luciferase, herpes simplex virus, post-Golgi carrier, Rab6, Rab6 effector, secretion, VSV-G

## Abstract

Herpes simplex virus 1 (HSV1) is an enveloped virus that uses undefined transport carriers for trafficking of its glycoproteins to envelopment sites. Screening of an siRNA library against 60 Rab GTPases revealed Rab6 as the principal Rab involved in HSV1 infection, with its depletion preventing Golgi-to-plasma membrane transport of HSV1 glycoproteins in a pathway used by several integral membrane proteins but not the luminal secreted protein *Gaussia* luciferase. Knockdown of Rab6 reduced virus yield to 1% and inhibited capsid envelopment, revealing glycoprotein exocytosis as a prerequisite for morphogenesis. Rab6-dependent virus production did not require the effectors myosin-II, bicaudal-D, dynactin-1 or rabkinesin-6, but was facilitated by ERC1, a factor involved in linking microtubules to the cell cortex. Tubulation and exocytosis of Rab6-positive, glycoprotein-containing membranes from the Golgi was substantially augmented by infection, resulting in enhanced and targeted delivery to cell tips. This reveals HSV1 morphogenesis as one of the first biological processes shown to be dependent on the exocytic activity of Rab6.

Intracellular trafficking between organelles is regulated by the Rab group of small GTPases 1. Specific Rabs are associated with distinct organelles and their associated transport vesicles, enabling accurate delivery of membranes and cargo to specific compartments. There are over 60 human Rabs which coordinate the processes of vesicle formation, transport, tethering and fusion, by interacting with specific effector proteins that bind to the GTP-bound form of their respective Rabs 2. In recent years a number of studies have provided growing evidence for the exploitation of Rabs by enveloped viruses in their morphogenesis and egress pathways. For example, influenza A and respiratory syncytial virus have both been shown to utilize the Rab11 pathway for envelopment and budding 3–5. Likewise, Rabs 7A and 9, both present on the membranes of late endosomes/multivesicular bodies, have been implicated in human immunodeficiency virus morphogenesis 6,7, while the early endosome localized Rab5 together with Rab7 are involved in the formation of hepatitis C virus replication complexes 8.

Herpes simplex virus 1 (HSV1) is a large enveloped DNA virus with a complex structure comprising up to 40 structural proteins 9. The genome-containing capsid is surrounded by the virus envelope containing up to 15 glycoproteins, and the tegument where over 20 virus encoded proteins are packaged 9,10. HSV1 is released from the infected cell following an intricate morphogenesis pathway that has been the subject of much debate 11,12. After capsid assembly in the nucleus, capsids are transported to the cytoplasm by first acquiring a primary envelope at the inner nuclear membrane that is lost by fusion with the outer nuclear membrane – termed an envelopment-deenvelopment step – releasing naked capsids into the cytosol 12,13. These free capsids acquire their tegument proteins and are re-enveloped at a site in the cytoplasm that remains a point of contention 12,14, with the favoured location of HSV1 envelopment commonly cited as the trans-Golgi network (TGN), where virus glycoproteins are known to localize 13,15,16. Virus production is sensitive to the endoplasmic reticulum (ER)-to-Golgi inhibitor Brefeldin A (BFA) 17,18, and is inhibited by the depletion of Rab1, a Rab known to be involved in ER-to-Golgi transport 19–22, suggesting that virus glycoproteins must access the Golgi/TGN prior to envelopment. Virus glycoproteins also localize to the plasma membrane (PM) in advance of any capsid accumulation in the cytoplasm 23–26.

Recently, we have published work providing evidence that, rather than wrapping at the TGN, HSV acquires its envelope from glycoprotein-containing endocytic membranes recently retrieved from the PM in a Rab5-dependent mechanism 22, in agreement with earlier studies from others 27. Depletion of Rab5 resulted in virus glycoproteins being trapped at the cell surface due to an inhibition of Rab5-dependent endocytosis, a subsequent failure in virus capsid wrapping and a 95% drop in virus yield. Hence, we suggest that virus envelope proteins are first transported through the Golgi/TGN to the PM ahead of capsid release from the nucleus, and are then retrieved into the recycling endocytic pathway to provide the final wrapping membranes. To identify additional Rabs involved in the HSV1 life cycle, we have now conducted an siRNA screen of 60 human Rabs to define their requirement in virus replication. The major factor that we have identified for production of infectious virus, over and above the aforementioned Rab1 or Rab5, is the Golgi-associated Rab6. In the absence of Rab6, HSV1 envelope proteins were unable to reach the cell surface and were retained in the Golgi/TGN during infection or when expressed in isolation. In infected cells, the retention of glycoproteins at the Golgi/TGN resulted in the subsequent accumulation of naked capsids which were not associated with infectivity. Notably, HSV1 infection induced the tubulation and redistribution of Rab6 positive membranes from the Golgi/TGN to peripheral membranes, delivering large amounts of envelope constituents to the PM in a pathway that requires the Rab6 effector ERC1 for optimal efficiency. Hence, we conclude that HSV1 activates a Rab6-specific exocytic pathway to transport virus glycoproteins from the Golgi/TGN to the PM, and provide a membrane population that is subsequently used for virus wrapping by endocytic retrieval. This reveals that the Rab6 specific post-Golgi pathway is fundamental to HSV1 envelopment.

## Results

### Depletion of Rab6 GTPase inhibits HSV1 virus production

**Figure 1:**
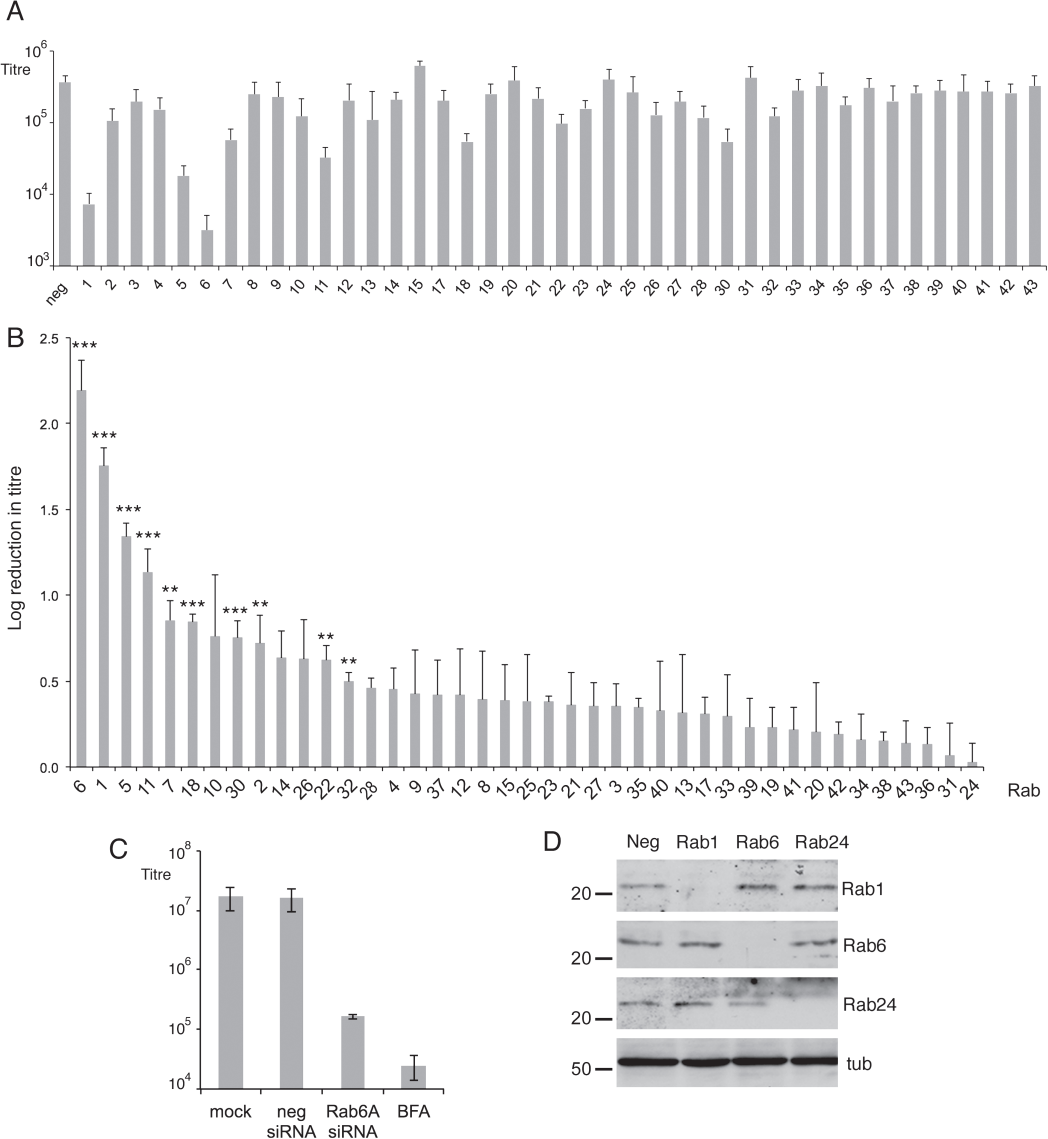
Requirement for human Rab GTPases in HSV1 infection. A) siRNAs from a library against 60 human Rabs were transfected into HeLa cells, alongside a control negative siRNA, pooling siRNAs to different isoforms of the same Rab. Two days later, the transfected cells were infected with HSV1 at a multiplicity of 2, and extracellular released virus harvested at 16 h and titrated on Vero cells. Error bars indicate standard error from three independent experiments. B) The graph from (A) is presented as the relative log drop in virus yield, in order from the largest to the smallest. Error bars indicate standard error from three independent experiments. Statistical analysis was carried out using the Student's *t*-test. ***p < 0.001; **p < 0.01. C) HeLa cells were transfected with negative or Rab6A siRNAs or left untransfected, and infected as above. Five hour after infection BFA (1 µg/mL) was added to one untransfected sample, and released virus harvested from all samples at 18 h. Error bars indicate standard error from three independent experiments. D) HeLa cells were transfected with the negative siRNA or pooled siRNAs to Rab1A and1B, Rab6A, 6B and 6C or Rab24 and analysed 2 days later by western blotting for these Rabs. Alpha tubulin (tub) was used as the loading control. Molecular weight markers are shown in kDa.

To investigate cellular transport pathways involved in HSV1 morphogenesis, we carried out a screen of human Rab GTPases using an siRNA library directed against the 60 Rabs in HeLa cells, a cell line commonly used for HSV1 infection. All siRNAs to individual isoforms of each Rab protein (2 duplexes per Rab) were pooled to make 41 groups of siRNAs, and transfected into HeLa cells, which were infected 48 h later with HSV1. Released virus was harvested from the cell media 16 h after infection and the resulting yields of each knockdown compared to that of the negative siRNA control. The average of three separate screens (Figure 1A) showed that depletion of only four Rabs – Rab1, Rab5, Rab6 and Rab11 – reduced virus yields by over 1 log, with depletion of Rabs 1 and 6 causing the greatest reduction of 53 and 130 fold, respectively. This is represented in Figure 1B as the relative log reduction in virus titre caused by the depletion of each Rab ranging from the Rab whose absence caused the largest drop (Rab6) to the one that caused the smallest drop (Rab24), showing that only a small number of Rabs caused a statistically significant reduction in virus production when depleted. Inhibition of virus replication by the drug BFA which blocks ER-to-Golgi transport – a treatment that has been shown to subsequently inhibit capsids leaving the nucleus 18 – reduced the level of virus in the extracellular media by around 500 fold, indicating the significance of a 130-fold drop by the depletion of only one cellular factor, Rab6 (Figure 1C). Of the Rab6 isoforms, Rab6A and its splice variant Rab6A'are known to be expressed ubiquitously in all tissues including HeLa cells, but Rab6B is neuronal specific, while Rab6C is expressed in only a limited range of human tissues 28,29. Moreover, transfection of siRNAs targeting only Rab6A had the same effect on virus yield as the combined siRNA pool against the three isoforms (Figure 1C), suggesting that Rab6A is the isoform important in these studies, and herein will be referred to as Rab6.

Rab1 is well-characterised as being involved in ER-to-Golgi transport and therefore would be considered to be fundamental for glycoprotein trafficking to the Golgi 19,20. However, it was notable that transfection of Rab6 siRNAs had a more profound effect on virus yield than transfection of Rab1 siRNAs. Western blots of cells transfected with siRNA for Rab1, Rab6 or Rab24, chosen as a control Rab that had no effect on virus yield on depletion, indicated that an efficient knockdown had been achieved in each case, confirming the efficacy of the siRNA sequences used (Figure 1D), with quantitative reverse transcriptase-polymerase chain reaction (RT-PCR) confirming the knockdown at around 97% for both Rab1 and Rab6 mRNA (Figure S1A, Supporting Information). Furthermore, cell viability measurements of Rab1 or Rab6 depleted cells indicated that HeLa cell viability was not affected by the absence of either these proteins (Figure S1B). Rab6 depletion also had the same effect on virus release from cells infected with two additional strains of HSV1 – Sc16 and HFEM – showing that the effect was independent of virus strain used (Figure S1C). In addition we carried out Rab6 knockdowns in two other human cell lines – the HaCaT and nTERT keratinocyte lines, cell types relevant to virus infection in the host – showing that Rab6 knockdown also inhibited virus production in cells other than HeLa (Figure S1D). Taken together, our results indicate that Rab6, and more specifically Rab6A, is a major determinant of HSV1 infection. This effect of Rab6 depletion on HSV1 replication is a new finding and the focus of this study.

### Rab6 depletion blocks virus production late in the infectious cycle

**Figure 2:**
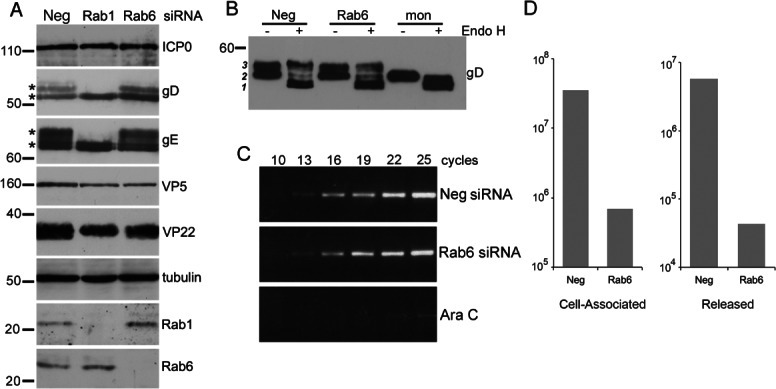
Rab6 depletion inhibits virus production at late stages of infection. A) HeLa cells transfected with neg, Rab1 or Rab6 siRNAs were infected with HSV1 2 days later and harvested 16 h after infection for western blotting with antibodies as indicated. Mature glycosylated forms of gD and gE are marked by asterisk. B) The status of glycoprotein glycosylation in control (neg), Rab6 depleted or monensin treated cells was determined by Endo H treatment of infected cell lysates harvested at 16 h, and analysed by western blotting for gD. Three isoforms of gD are denoted by *1, 2 & 3*. C) The level of DNA replication in control (neg), Rab6 depleted or Ara C treated cells was analysed by semi-quantitative PCR using a primer pair specific for the UL47 gene. PCR cycle numbers are denoted. D) Hela cells transfected with neg or Rab6 siRNAs were infected 2 days later with HSV1, and cell-associated and released virus titrated on Vero cells. Molecular weight markers are shown in kDa.

To further assess the effect of Rab6 depletion on the HSV1 infection process, we first examined the level of several virus proteins present in infected cells by western blotting of lysates harvested 16 h after infection. Importantly, the levels of proteins tested for, including the immediate-early protein ICP0, and the late structural proteins VP5 and VP22, were unaffected in either Rab1 or Rab6 knockdowns, confirming that these virus proteins accumulated appropriately in depleted cells and there was no apparent inhibition of early stages of virus infection in these cells (Figure 2A). Western blotting of glycoproteins gD and gE indicated that the mature glycosylated forms of these proteins were greatly reduced in Rab1 depleted cells, as shown by others 21, but were present at normal levels in Rab6 depleted cells. As proteins are terminally glycosylated by Golgi resident enzymes 30, Rab1 depletion which inhibits ER-to-Golgi transport would be expected to inhibit the full glycosylation of these proteins. Thus, as the gD and gE glycoprotein profiles are the same in control and Rab6 depleted cells we would predict that virus glycoproteins have been successfully transported from the ER to the Golgi in the absence of Rab6. To confirm that gD in Rab6 depleted cells is fully mature, we treated cell lysates with endoglycosidase H (Endo H), an enzyme that cleaves N-linked high mannose oligosaccharides, but not fully processed complex oligosaccharides from glycoproteins. Treatment of control gD resulted in the disappearance of the lower molecular weight gD form ***2***, and the appearance of a faster migrating form ***1*** of gD, representing the de-glycosylated protein (Figure 2B). While a proportion of the upper molecular weight gD form ***3*** had also been cleaved, a significant amount remained intact, thus representing the fully processed, mature, glycoprotein. The same treatment of gD in Rab6 depleted cells resulted in a similar profile, suggesting gD is processed in the same way in the absence of Rab6, and hence Rab6 is not involved in the transit of gD through the Golgi. Interestingly, monensin treatment of infected cells, a drug which blocks protein transport from the Golgi to the PM 31, inhibited the production of the upper molecular weight form ***3*** of gD in a manner similar to Rab1 depletion (Figure 2B), suggesting that monensin may either block gD transport through the Golgi at a stage prior to final processing, or may affect the localization of the enzymes involved. This provides further evidence that the absence of Rab6 does not adversely affect the glycosylation of virus envelope proteins.

To ensure that Rab6 depletion did not inhibit virus genome replication, we next measured the level of HSV1 genomic DNA in Rab6 depleted cells compared to control siRNA transfected cells or cells treated with the DNA replication inhibitor Ara C. Total DNA harvested from infected cells was subjected to semi-quantitative PCR using a primer pair that amplifies a 300 bp fragment of the UL47 gene 32. Under these conditions, PCR product was first detectable after 13 PCR cycles in control and Rab6 depleted cells, and amplified at a similar rate, while in Ara C treated cells it was only detectable after 25 cycles (Figure 2C). Hence, virus DNA replication proceeds normally in the absence of Rab6.

Because the depletion of Rab6 had little effect on virus protein expression and DNA replication, and the original siRNA knockdown screens had measured only released virus, it remained a possibility that virus was assembling correctly in the cell but was unable to undergo egress and release from the cell. To assess this, we compared the reduction in cell-associated infectivity in the absence of Rab6 to the reduction in released infectivity in the absence of Rab6 (Figure 2E). This demonstrated a close to 2 log reduction in cell-associated virus in the absence of Rab6, confirming that virus morphogenesis *per se* is the likely block in Rab6 depleted cells.

### Rab6 depletion inhibits HSV1 glycoprotein trafficking from the Golgi to the PM

**Figure 3:**
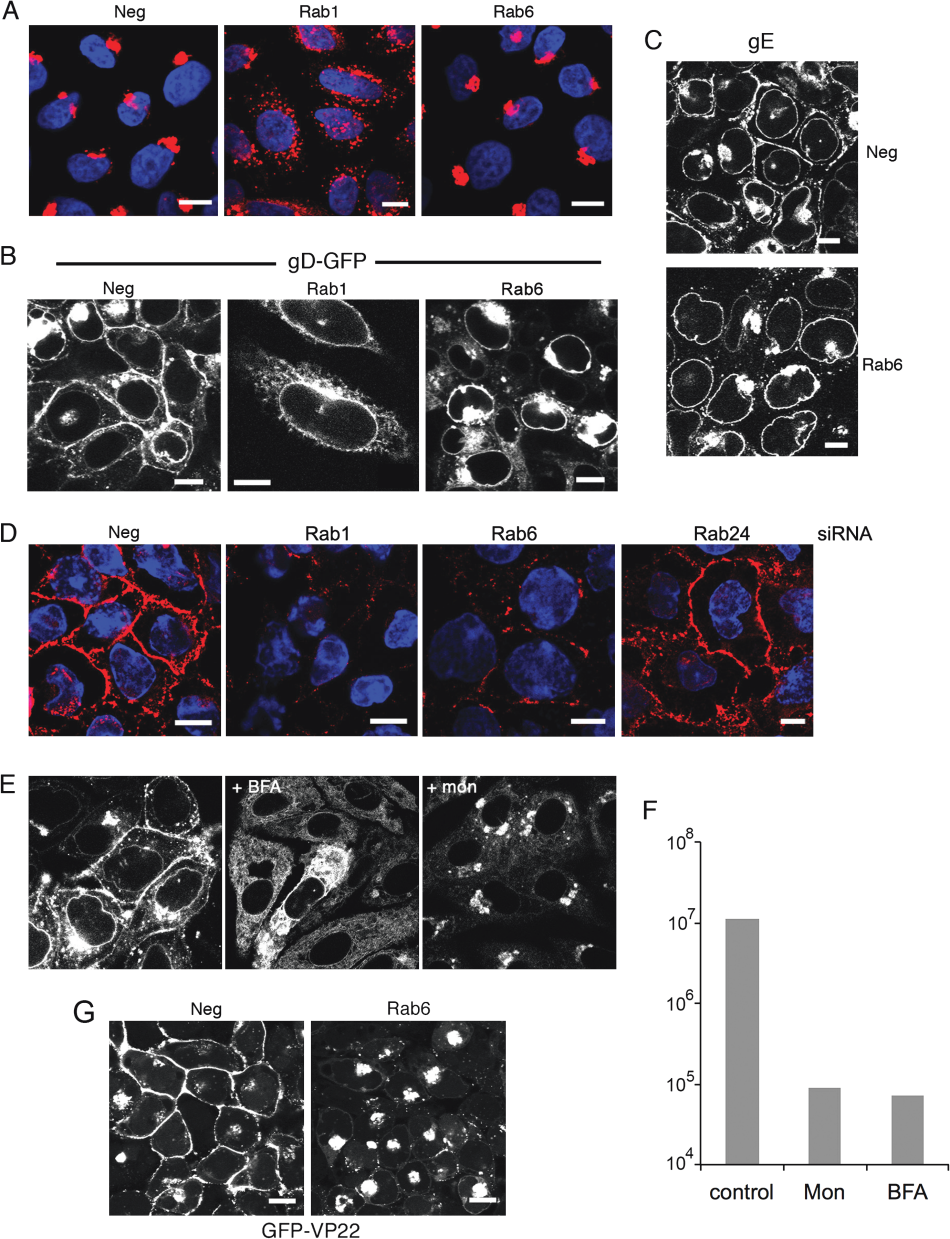
Rab6 depletion inhibits HSV1 glycoprotein localization to the plasma membrane in infected cells. A) HeLa cells grown on coverslips were transfected with neg, Rab1 or Rab6 siRNAs, fixed 2 days later and stained for giantin (red), and nuclei stained wih DAPI (blue). B) HeLa cells on coverslips transfected with neg, Rab1 or Rab6 siRNAs were infected 2 days later with HSV1 expressing gD-GFP and fixed 16 h later. C) Neg or Rab6 siRNA transfected HeLa cells were infected after 2 days with Wt HSV1 and stained with a monoclonal antibody against gE. D) HeLa cells on coverslips were transfected with neg, Rab1, Rab6 or Rab24 siRNAs and infected 2 days later with Wt HSV1. Sixteen hours later the cells were incubated on ice for 30 min with gD monoclonal antibody to detect cell surface protein, washed and fixed prior to staining with secondary antibody (red). Nuclei were stained with DAPI (blue). E) Cells infected with HSV1 expressing gD-GFP at a multiplicity of 2 were treated 5 h later with BFA, monensin or left untreated. Cells were fixed at 12 h. F) Total virus yield from infections carried out as for (E), and harvested 18 h after infection. G) HeLa cells on coverslips were transfected with neg or Rab6 siRNA, infected 2 days later with HSV1 expressing the tegument protein VP22 as a GFP fusion protein (GFP-VP22), and images acquired after 16 h. Scale bar = 10 µm.

Rab6 localizes to the trans-Golgi cisternae/TGN and has been implicated in several trafficking pathways originating at the Golgi, including intra-Golgi retrograde trafficking, COPI independent retrograde transport to the ER, and anterograde transport from the Golgi/TGN to the PM 33–37. As shown above, Rab6 does not appear to be required for the maturation of virus glycoproteins. Staining of uninfected cells for the Golgi-localized membrane protein giantin indicated that while the Golgi was markedly fragmented in Rab1 knockdown cells (Figure 3A, Rab1), in Rab6 depleted cells giantin localization was unaltered (Figure 3A, Rab6). Hence, unlike Rab1 depletion, Rab6 depletion had no obvious effect on the Golgi, as shown previously 38,39. Examination of HeLa cells infected with HSV1 expressing green fluorescent protein (GFP) fused to the glycoprotein gD (gD-GFP) revealed that by 14 h after infection in untreated cells, gD was detected in abundance at the PM and to a lesser extent in the nuclear membrane, while in Rab1 depleted cells it was localized predominantly in an ER pattern and was absent from the PM (Figure 3B). In Rab6 depleted cells gD accumulated in a juxtanuclear Golgi/TGN location, was heavily concentrated in the nuclear membrane, and failed to accumulate at the PM (Figure 3B). This failure to localize to the PM was evident as late as 20 h after infection (Figure S2). Immunofluorescence of gE confirmed that this lack of glycoprotein localization at the cell surface and enhanced nuclear membrane staining in Rab6 depleted cells was not restricted to gD-GFP (Figure 3C). The lack of PM-localized glycoprotein in both Rab1 and Rab6 depleted cells was confirmed by cell surface staining with an antibody specific for the extracellular domain of gD (Figure 3D). In negative and Rab24 depleted cells, gD antibody bound efficiently to the cell surface confirming the presence of gD in the PM, but in the Rab1 and Rab6 depleted cells gD antibody binding to the cell surface was minimal. Interestingly, gD-GFP localization in Rab1 and Rab6 depleted cells broadly reflected its localization when infected cells were treated with either the ER-to-Golgi inhibitor BFA or the Golgi-to-PM inhibitor monensin 31, respectively (Figure 3E), treatments that also inhibited virus production (Figure 3F). However, for an as yet unknown reason, monensin treatment inhibited the appearance of gD-GFP in the nuclear membrane. These data suggest that Rab1 and Rab6 are fundamental for different stages of HSV1 glycoprotein trafficking to the PM in infected cells – ER-to-Golgi in the case of Rab1, and Golgi-to-PM in the case of Rab6.

In light of our results showing failed glycoprotein trafficking to the PM in Rab6 depleted cells, we next investigated the effect of Rab6 depletion on the trafficking of the tegument protein VP22, a protein that is known to be assembled into the virus by interacting with the cytoplasmic tails of the glycoproteins gE and gM 40. HeLa cells transfected with negative or Rab6 siRNAs were infected 2 days later with HSV1 expressing GFP-tagged VP22 41, and analysed 16 h after infection. In control cells, GFP-VP22 was localized predominantly at the PM at this time (Figure 3G). However, in Rab6 depleted cells, GFP-VP22 was clearly mislocalized to a juxtanuclear Golgi-like location (Figure 3G), suggesting that like the glycoproteins, the trafficking of this tegument protein was affected by the lack of Rab6, a feature that was evident as late as 20 h after infection (Figure S3). Such abnormal VP22 concentration around the Golgi in the absence of Rab6 may suggest that this is the site of VP22 recruitment to the virus envelope, but that in a normal infection, it is rapidly transported to the PM.

### Rab6 depletion inhibits capsid association with wrapping membranes

**Figure 4:**
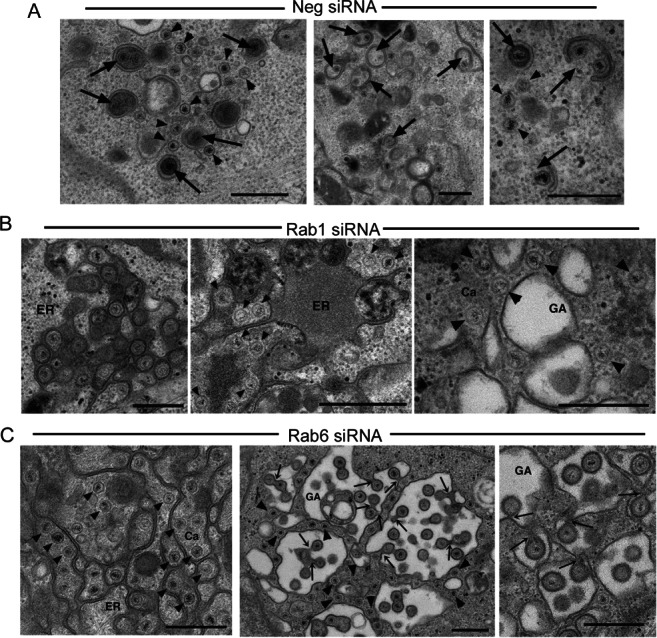
Rab6 depletion inhibits HSV1 capsid envelopment. Hela cells transfected with (A) neg, (B) Rab1 or (C) Rab6 siRNAs were infected 2 days later with HSV1, fixed at 16 h and processed for transmission electron microscopy. ER, endoplasmic reticulum; GA, Golgi apparatus; Ca, capsid. Arrowheads indicate naked capsids. Thick arrows indicate capsids associated with wrapping membranes. Thin arrows indicate continuum with the cytoplasm. Scale bar = 500 nm.

The above results suggest that the block in virus production in the absence of Rab6 occurs late in infection, but prior to virus morphogenesis. We have recently published a detailed ultrastructural analysis of HSV1 morphogenesis defining various stages of HSV1 assembly that we observe during infection 22, showing that capsids are wrapped by endocytic tubular membranes at locations throughout the cytoplasm of the infected cell. Moreover, we showed that inhibition of endocytosis blocked the appearance of these profiles, and instead capsids associated aberrantly with membranes of the Golgi. Using this framework for determining the state of virus morphogenesis in our studies here, we carried out electron microscopy on infected HeLa cells (at 12 h) that had been transfected with negative, Rab1 or Rab6 siRNAs. In control infected HeLa cells capsids were readily found in association with wrapping membranes (Figure 4A, thick arrows) as we have described before 22. By contrast, in Rab1 depleted cells, although cytoplasmic capsids were detected leaving the ER, the majority of capsids were unwrapped and were detected associating with the membranes of the rough ER, and interspersed between membranes of dilated Golgi (Figure 4B, arrowheads). Likewise, in Rab6 depleted cells, multiple naked capsids were found in association with the rough ER and Golgi membranes (Figure 4C, arrowheads). Moreover, in some locations, a proportion of capsids at the Golgi gave the appearance of attempting to bud into the cisternae of these membranes (Figure 4C). Close examination of these profiles revealed that no intact virions were obviously present in the lumina, but instead the capsids were connected to the surrounding cytoplasm by short, cytoplasm-containing, stalks (Figure 4C, thin arrows). These profiles are strikingly similar to our previous observations of failed budding events in Rab5 depleted cells where, unlike here, we have shown that virus glycoproteins are not restricted in Golgi/TGN to PM transport, but instead as described above, are blocked from being re-endocytosed into the endocytic network 22. Because of those recent findings showing that endocytosis of virus glycoproteins from the PM is required for virus envelopment, we also tested the effect of Rab6 depletion on the uptake of transferrin by endocytosis. HeLa cells transfected with negative or Rab6 specific siRNAs were incubated with texas red-transferrin for 30 min, fixed and counterstained for Rab6 (Figure S4). This confirmed that the absence of Rab6 had no effect on the endocytic uptake of transferrin and hence did not inhibit clathrin mediated endocytosis. This suggests that the defective Golgi localization of capsids – which is not observed in normal infection but is observed in both Rab5 and Rab6 depleted cells – is a consequence of capsid mislocalization in the absence of authentic endocytic wrapping membranes. Taken together, these results suggest that in the absence of Rab6, virus proteins are synthesized, capsids are assembled in the nucleus, and traffic to the cytoplasm. Nonetheless, infectious particle production is blocked, correlating with the mislocalization of glycoproteins and at least one tegument protein at the Golgi/TGN, a feature that is also reflected in the capsid mislocalization observed by electron microscopy. Importantly, this implies that glycoprotein trafficking to the Golgi/TGN is not sufficient for the production of infectious virus.

### Rab6 is redistributed to the cell periphery in HSV1 infected cells

**Figure 5:**
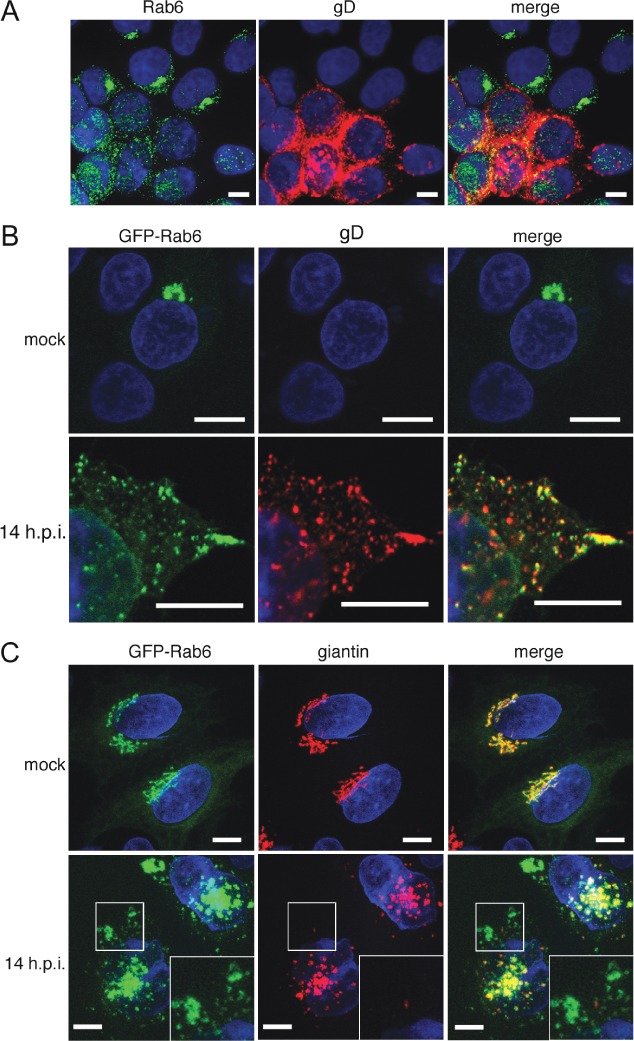
Rab6 is redistributed and colocalizes with HSV1 glycoproteins at the cell periphery. A) HeLa cells infected with HSV1 at a multiplicity of 0.5 were fixed 16 h later and stained for endogenous Rab6 (green) and gD (red). Nuclei were stained with DAPI (blue). B and C) HeLa cells transfected with plasmid expressing GFP-Rab6 (green) were mock or HSV1 infected, fixed 14 h later and stained with antibodies for the glycoprotein gD (B) or the Golgi marker giantin (C) (red). Nuclei were stained with DAPI (blue). Scale bar = 10 µm.

The requirement for Rab6 in HSV1 replication led us to examine its localization during HSV1 infection. Immunofluorescence of endogenous Rab6 in infected HeLa cells indicated that Rab6 was relocalized from the Golgi/TGN towards the cell periphery of infected cells (Figure 5A), while western blotting indicated no increase in Rab6 expression in HSV1 infected cells (data not shown). Although Rab6 is known to associate with vesicles derived from the Golgi/TGN that are destined for the cell surface 36, the majority of it is generally associated with the Golgi/TGN. To look in more detail at the location of Rab6 in infected cells, it was found necessary to express exogenous GFP-Rab6 42, because the Rab6 antibody available to us was not sensitive enough to detect endogenous protein with clarity. GFP-Rab6 localized to the Golgi/TGN in uninfected cells but was relocated to the cell periphery in infected cells, reflecting the distribution of endogenous Rab6 (Figure 5B). Further costaining of GFP-Rab6 expressing, HSV1 infected, cells with a gD antibody showed that a high percentage of GFP-Rab6 peripheral vesicles – quantitated at 80% – were also positive for this glycoprotein (Figure 5B, 14 h.p.i.) and others that we have tested (data not shown). Costaining of GFP-Rab6 expressing cells with the Golgi marker giantin, revealed that in uninfected cells these two proteins were mainly co-localized, and although GFP-Rab6 and giantin staining were both fragmented in infected cells, reflecting the known virus-induced Golgi fragmentation 22,43, a large population of GFP-Rab6 in the outer part of the cell did not costain for giantin (Figure 5C). These data imply that a substantial proportion of glycoprotein-containing membrane structures leaving the Golgi/TGN may also have Rab6 associated with them.

### Rab6 is differentially involved in secretion

**Figure 6:**
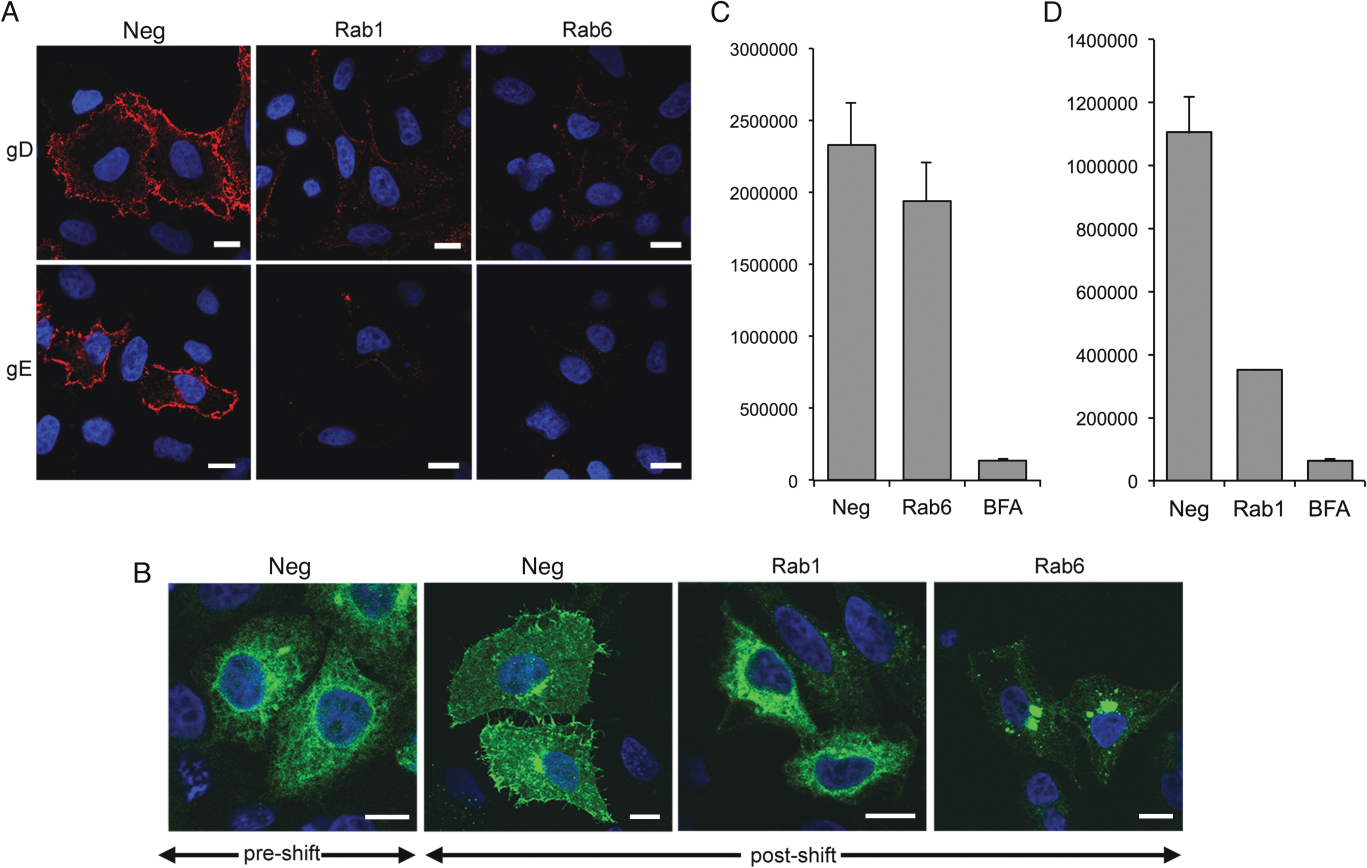
Rab6 depletion inhibits trafficking of gD, gE and tsVSV-G, but not *Gaussia* luciferase. A) HeLa cells on coverslips were transfected with neg, Rab1 or Rab6 siRNAs and 2 days later transfected with plasmids expressing gD or gE. After 16 h the cells were processed for cell surface staining with the appropriate antibody, incubated with secondary antibody (red), and stained with DAPI (blue). B) HeLa cells on coverslips were transfected with neg, Rab1 or Rab6 siRNAs and 2 days later transfected with plasmid expressing GFP-tsVSV-G protein (green). The cells were incubated overnight at the non-permissive temperature of 39°C (pre-shift), then incubated at the permissive temperature of 32°C for 4 h (post-shift) before fixation and staining with DAPI (blue). C) HeLa cells treated as indicated were transfected with a plasmid expressing *Gaussia* luciferase and incubated for 16 h. Media was changed and 1 h later the level of secreted *Gaussia* luciferase was measured, with the amount secreted from cells transfected with the neg siRNA taken as 1. As a positive control BFA was added to cells at a concentration of 5 µg/mL before assay. Un, untransfected cells. Error bars indicate standard error from three experiments. Scale bar = 10 µm.

As our previous results on HSV1 glycoprotein trafficking were obtained in the specialized environment of the infected cell, we next investigated the effect of Rab6 depletion on the trafficking of gD or gE expressed in isolation by transient transfection. In control transfected HeLa cells, both glycoproteins were detected on the cell surface (Figure 6A, neg), whereas glycoprotein levels at the cell surface were greatly reduced in Rab1 depleted cells, as expected from its role in ER-to-Golgi transport (Figure 6A, Rab1). In Rab6 depleted cells, the level of both glycoproteins at the cell surface was also greatly reduced (Figure 6A, Rab6) confirming that Rab6 is required for the inherent trafficking pathway of these HSV1 glycoproteins to the cell surface.

To further define the specificity of Rab6 specific transport pathways to the PM, we next examined the trafficking of two reporter proteins in Rab6 depleted cells, using Rab1 depletion as a positive control. We first tested the role of Rab6 in the transport of the model cargo protein tsVSV-G protein from the ER to the PM. Following transfection with negative, Rab1, or Rab6 siRNAs, HeLa cells were transfected with a plasmid expressing GFP-tsVSV-G and incubated overnight at the non-permissive temperature of 39°C. Cells were then moved to the permissive temperature of 32°C and fixed 4 h later to examine the localization of GFP-tsVSV-G. In cells left at 39°C, GFP-tsVSV-G was localized in the ER as expected (Figure 6B, neg, pre-shift). After incubation at 32°C, GFP-tsVSV-G localized to the Golgi and the cell surface in cells transfected with the negative siRNA (neg, post-shift), but remained ER-localized in Rab1 siRNA transfected cells, reflecting the role of Rab1 in ER-to-Golgi transport (Figure 6B, Rab1, post-shift). As expected, Rab6 depletion had no effect on movement of GFP-tsVSV-G from the ER to the Golgi, but further transport to the PM was greatly reduced, suggesting a role for Rab6 in tsVSV-G movement away from the Golgi (Figure 6B, Rab6, post-shift). While, this is in agreement with other studies where it has been shown that the transport of tsVSV-G to the cell surface is significantly delayed or inhibited in the absence of Rab6 36,44, another recent study has suggested that Rab6 is not present in VSV-G Golgi-to-PM vesicles 45.

We also tested the secretion of the luminal *Gaussia* luciferase (GLuc) from HeLa cells that had been initially transfected with negative, Rab1 or Rab6 siRNAs. As a positive control, GLuc expressing cells were also treated with BFA for 4 h to inhibit transport from the ER to Golgi. As expected BFA inhibited the secretion of GLuc into the extracellular media (Figure 6C). Likewise, Rab1 depletion inhibited GLuc secretion, confirming that Rab1 is involved in the secretion of GLuc (Figure 6C). By contrast, Rab6 depletion had little effect on the level of secreted GLuc (Figure 6C), suggesting that unlike the HSV1 glycoproteins and the tsVSV-G protein, Rab6 is not involved in the secretion pathway of GLuc. These results indicate a differential involvement of Rab6 in secretion of reporter proteins, and suggest the existence of a specific, Rab6-dependent, Golgi-to-PM pathway required for HSV1 glycoprotein targeting to the cell surface.

### The role of Rab6 effectors in HSV1 production

**Figure 7:**
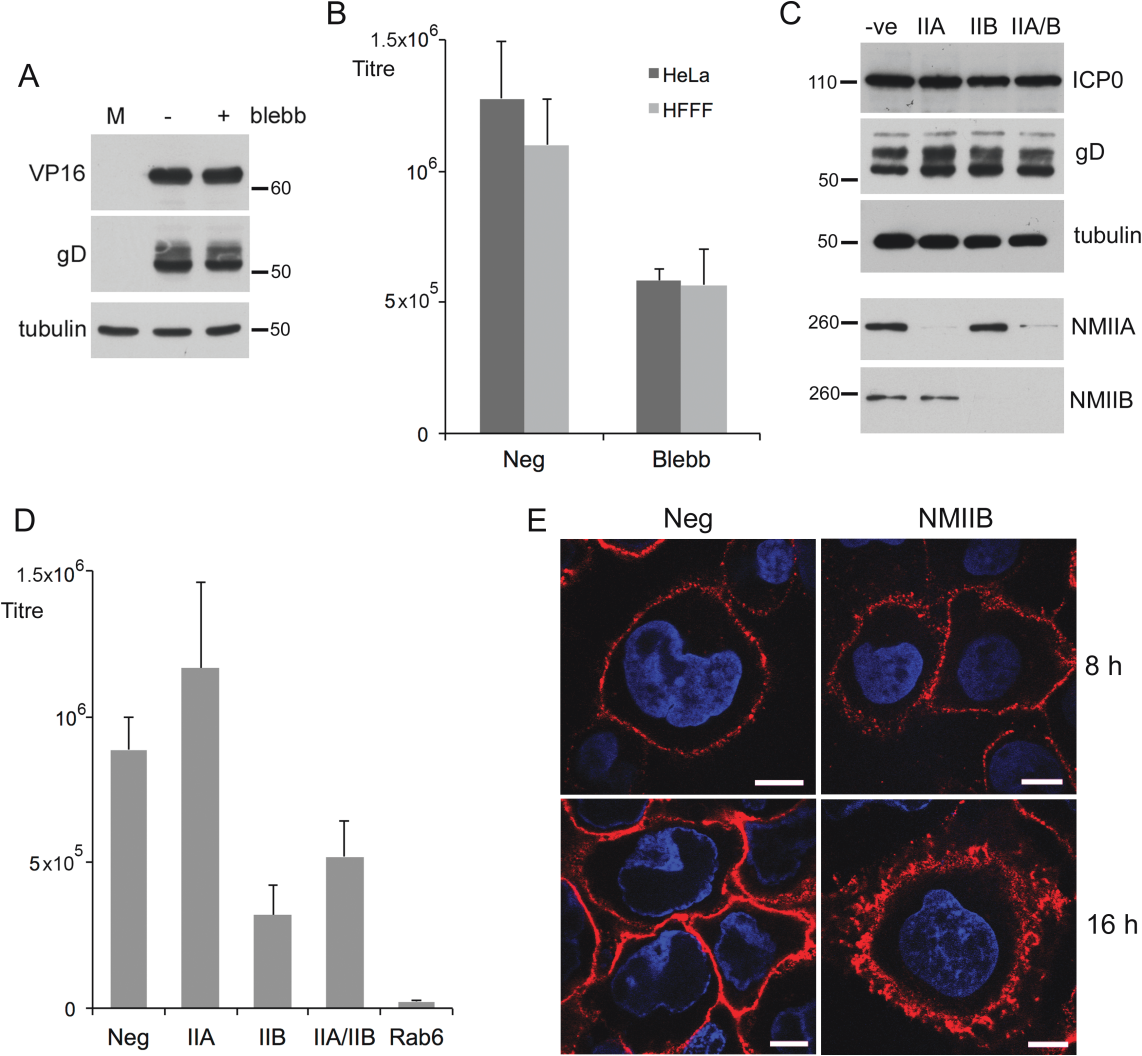
The Rab6 fission effector non-muscle myosin II is not required for HSV1 morphogenesis. A) HeLa cells infected with HSV1 at a multiplicity of 2 were treated with blebbistatin at 5 h or left untreated and harvested for western blotting at 16 h. B) HeLa or HFFF cells infected with HSV1 at a multiplicity of 2 were treated with blebbistatin at 5 h or left untreated and harvested for extracellular virus yield at 18 h. C and D) HeLa cells were transfected with neg, myosin IIA (IIA) or myosin IIB (IIB) siRNAs, infected 2 day later with HSV1 at a multiplicity of 2, and harvested for western blotting (C) or extracellular virus (D). E) HeLa cells on coverslips were transfected with neg or myosin IIB (NMIIB) siRNAs and infected 2 days later with Wt HSV1. Eight and sixteen hours later the cells were processed for gD detection at the cell surface, and stained with secondary antibody (red). Nuclei were stained with DAPI (blue). Scale bar = 10 µm. Error bars indicate standard error from three experiments. Molecular weight markers are shown in kDa.

To refine the mechanism of HSV1 glycoprotein trafficking, and the role of Rab6 in this process, we investigated the requirement for a range of Rab6 effectors in the Golgi-to-PM transport of glycoproteins. Fission of Rab6 vesicles from the Golgi/TGN has been shown to be promoted by non-muscle myosin heavy chain II (NMHCII) 37, a factor that has also been implicated in HSV1 morphogenesis 46. However, treatment of HSV1 infected HeLa cells with the specific myosin inhibitor blebbistatin 6 h after infection resulted in only a two-fold reduction in virus production (Figure 7B, HeLa) while having no effect on protein levels (Figure 7A). Blebbistatin treatment of HSV1 infection of a different cell line, the primary human fibroblast line HFFF, resulted in the same twofold drop in virus yield (Figure 7B, HFFF). Depletion experiments were also carried out by transfecting HeLa cells with siRNAs specific for NMHCIIA, NMHCIIB or both together. Depletion of both proteins was efficient and had little effect on virus protein levels (Figure 7C). However, while virus yield remained unaffected when NMHCIIA was depleted, depletion of NMHCIIB alone or in combination with NMHCIIA, resulted in a modest but reproducible twofold drop in virus yield (Figure 7D). Localization of gD to the cell surface in cells depleted of NMHCIIB was similar to cells transfected with the neg siRNA at both early and late times, although the appearance of the PM was markedly different (Figure 7E). In short, the Rab6 fission effector myosin II is not fundamental to the transport of virus glycoproteins to the PM or HSV1 production.

**Figure 8:**
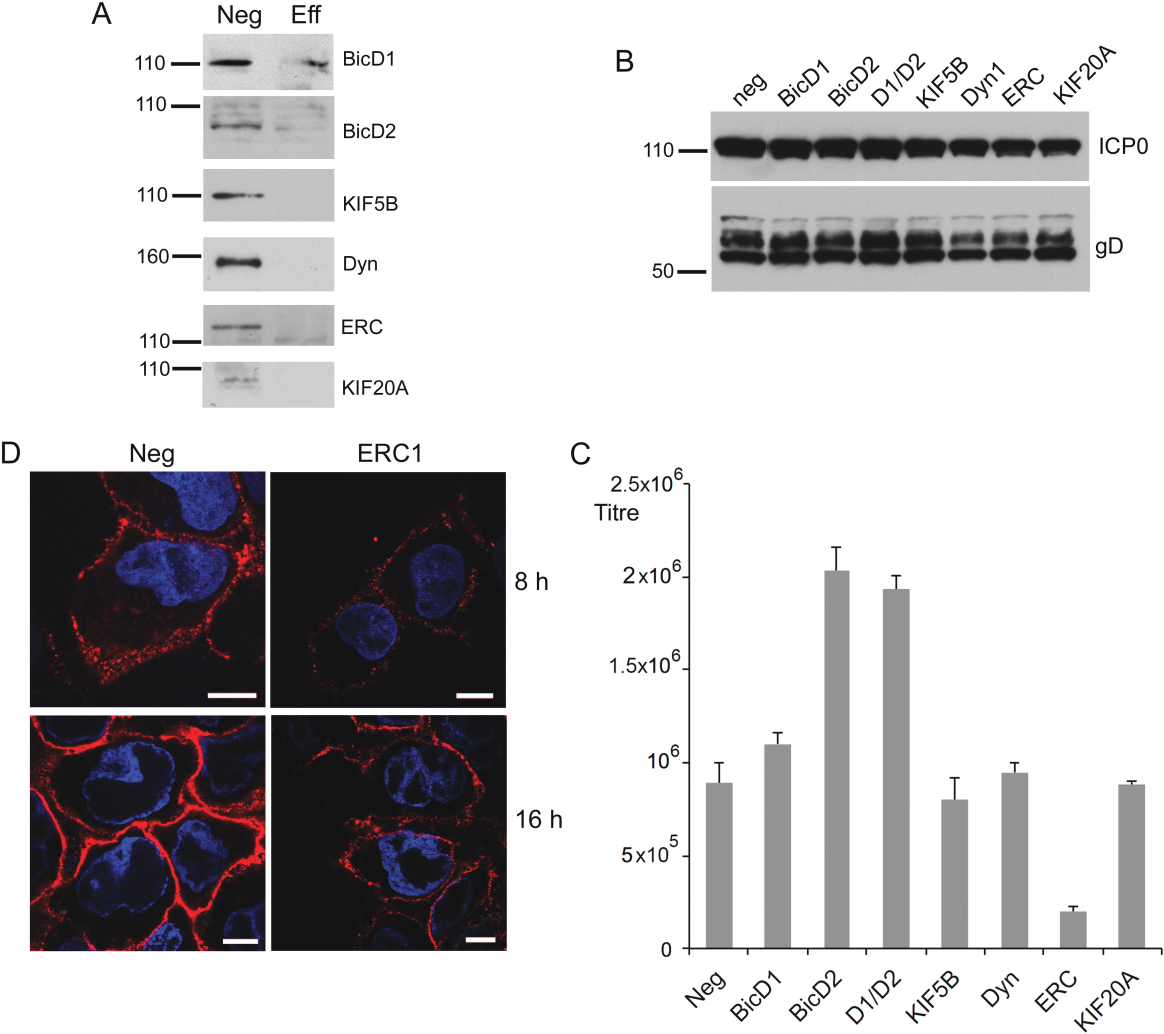
The Rab6 effector ERC1 facilitates HSV1 glycoprotein trafficking and morphogenesis. A–C) HeLa cells were transfected with neg siRNA or siRNA to bicaudal D1 (BicD1), bicaudal D2 (BicD2), conventional kinesin heavy chain (KIF5B), dynactin 1 (Dyn), ERC1 or rabkinesin 6 (KIF20A) and 2 days later were either harvested for western blotting with antibodies specific to each effector (A) or infected with HSV1 at a multiplicity of 2, and harvested at 16 h for western blotting for the virus proteins ICP0 and gD (B), or extracellular virus (C). Error bars indicate standard error from three experiments. D) HeLa cells on coverslips were transfected with neg or ERC1 siRNAs and infected 2 days later with HSV1. Eight or sixteen hours later the cells were processed for detection of cell surface gD, and stained with secondary antibody (red). Nuclei were stained with DAPI (blue), and images were acquired with the same acquisition settings. Scale bar = 10 µm. Molecular weight markers are shown in kDa.

These data raised the possibility that other cellular Rab6 effectors may be involved in HSV1 glycoprotein trafficking, and to this end we screened a range of siRNAs to other known effectors – the golgins bicaudal-D1 and -D2 42 depleted alone or in combination; the dynactin subunit DCTN1 47; the kinesin family member rabkinesin-6, also known as KIF20A 48; and the cortical protein ERC1, also known as ELKS 49. In addition, we included the conventional kinesin heavy chain encoded by KIF5B in the screen, as this has been shown to play a role in the movement of Rab6 positive vesicles on microtubules 36,37. Carried out as previously, and in spite of efficient knockdown (Figure 8A), the screen showed that of all these effectors, ERC1 was the only one that, when depleted, caused a demonstrable and reproducible drop in HSV1 yield, reducing it by 5–10 fold (Figure 8C), without affecting the level of virus proteins in infected cells (Figure 8B). This lower virus production correlated with a reduction in the level of gD at the cell periphery in ERC1 depleted cells which was apparent at both early and late times (Figure 8D). Interestingly, the depletion of bicaudal D2, but not D1, resulted in a reproducible 2-fold increase in virus yield. Surprisingly, neither the dynactin subunit DCTN1, nor the conventional kinesin heavy chain KIF5B were required for HSV1 production in infected HeLa cells, as their efficient depletion had no effect on virus yield. Moreover, depletion of rabkinesin-6, a Golgi localized Rab6 effector, had little effect on virus yield, in spite of causing the formation of large multinucleate cells (data not shown), presumably a consequence of its essential role in cytokinesis 50.

### Enhanced tubulation of GFP-Rab6 positive membranes in infected cells

**Figure 9:**
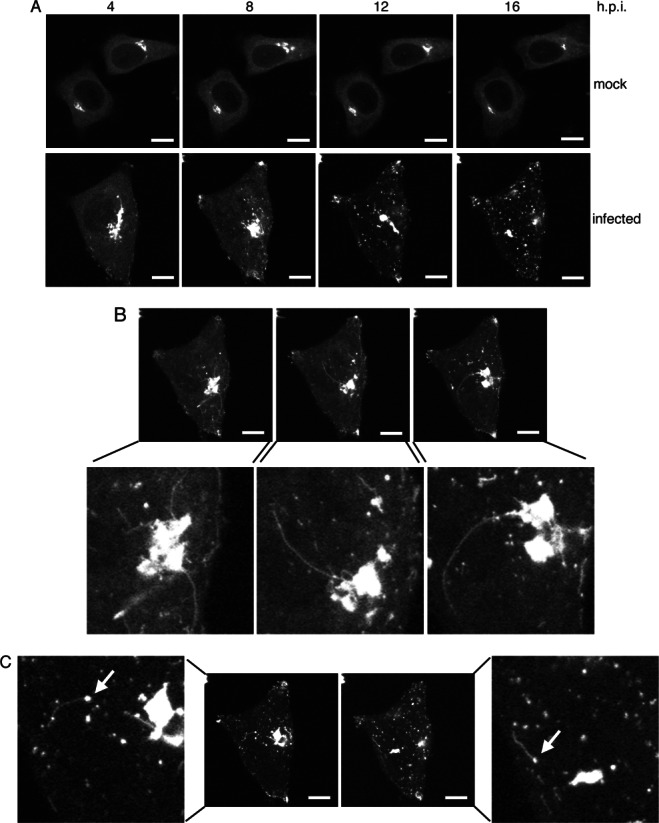
Rab6 trafficking to the cell surface is activated in HSV1 infected cells. HeLa cells were transfected with plasmid expressing GFP-Rab6 and left uninfected (mock) or infected 4 h later with HSV1 (infected). After a further 4 h, time-lapse analysis was carried out with images acquired every 5 min for 16 h. A) Images acquired at 4 hourly intervals through the course of the time-lapse showing the progressive dispersal of GFP-Rab6 through infection. B and C) Representative images showing GFP-Rab6 positive tubules emanating from the area of the Golgi/TGN (B) or GFP-Rab6 positive domains in cytoplasm (C). Arrows indicate peripheral sites from where tubules extend. Scale bar = 10 µm. See also Video S1.

The redistribution of Rab6 in HSV1 infected cells implied that the characteristics of Rab6-dependent trafficking may be altered in HSV1 infected cells. To determine the dynamics of GFP-Rab6 trafficking in infected cells, we carried out time-lapse confocal microscopy on mock or infected HeLa cells expressing GFP-Rab6 by transient transfection. Cells expressing low levels of GFP-Rab6 were deliberately chosen in an attempt to reflect endogenous levels of Rab6 and avoid issues with over-expression. Changes in GFP-Rab6 localization were analysed over a period of 12 h, with images initially being acquired at 5 min intervals. Rab6 Golgi localization in uninfected HeLa cells was consistent throughout such a time period (Figure 9A, mock). By contrast, the localization of Rab6 in HSV1 infected cells changed dramatically over this 12 h period (Figure 9A, infected). At the start of the animation (4 h after infection) GFP-Rab6 was predominantly localized to the Golgi/TGN, but there was already some evidence of Rab6 at the cell periphery (Figure 9A and Video S1). Over the 12 h time period, Rab6 became concentrated at the tips of cells, in addition to translocating from a juxtanuclear location to smaller domains throughout the cytoplasm (Figure 9A and Video S1). Additionally, in various frames of the animation, long tubules were seen to emanate from the Golgi towards the cell periphery (Figure 9B), which at later times also extended from the smaller GFP-Rab6 positive domains (Figure 9C).

**Figure 10:**
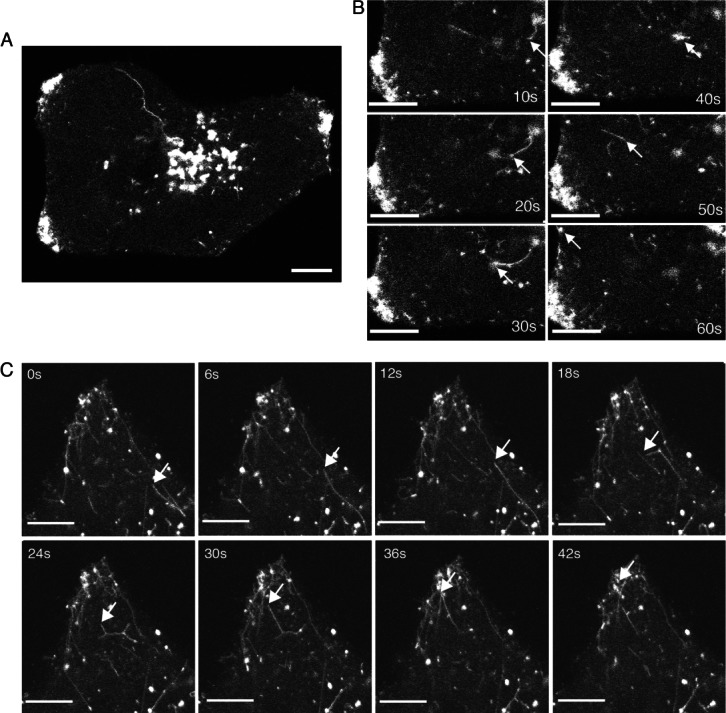
Enhanced exocytosis of Rab6 tubules in HSV1 infected cells. A and B) HeLa cells were transfected with plasmid expressing GFP-Rab6 and infected 12 h later with HSV1 at a multiplicity of 2. After 8 h (A and B) or 16 h (C), images were acquired at (B) 10 seconds or (C) 1 seconds intervals. Scale bar = 10 µm. See also Videos S2–S4.

The tubulation events in infected cells were examined more closely by imaging cells at 10 second intervals for a time period of 30 min (Figure 10A, Videos S3,S4). Under these conditions it was possible to track Rab6 positive tubules leaving the Golgi/TGN, trafficking towards the cell surface, and accumulating at the cell periphery (Figure 10B). By contrast, tubules were not observed leaving the Golgi in uninfected cells (Video S2). More frequent acquisition, with frames taken every second clearly showed the rate of movement of these tubules and on occasions revealed branching of tubules on their way to the cell surface (arrowed in Figure 10C and Video S5). In summary, our results show that HSV1 utilizes Rab6 specific Golgi-to-PM transport carriers for delivery of its envelope proteins to the cell surface, a process it appears to activate for efficient and timely onward trafficking to sites of virus envelopment.

## Discussion

In this study we present the first screen of human Rab GTPases in a virus infection of human cells. After screening sixty Rabs, we found that the depletion of only four pools reduced HSV1 yield by over 10 fold – in increasing order of effect from Rab11 (12.5 fold), Rab5 (21 fold), Rab1 (53 fold) to Rab6 (130 fold). Of these four, Rabs 1, 5 and 11 have been identified previously by us and others 21,22, but the conclusion that Rab6 is an important factor in HSV1 morphogenesis is a new finding. Rab6 is the major Rab GTPase associated with the trans cisternae of the Golgi and the TGN 51. It has been shown to play several roles in membrane trafficking, being identified as having a role in COP1-independent retrograde trafficking from the Golgi to the ER of certain proteins such as Golgi glycosylation enzymes and Shiga toxin 33. Additionally Rab6 is believed to be involved in retrograde intra-Golgi trafficking 52. However, in recent years there have been a number of studies invoking a role for Rab6 in vesicular trafficking from the Golgi/TGN to the PM 36,37,53, a specific role that would fit with our own findings.

Within the TGN, cargos are sorted into tubules away from resident Golgi proteins, and motors such as kinesin dock onto these tubules which then undergo extrusion along MTs, before fission from the Golgi/TGN occurs to form post-Golgi carriers (PGCs) 54. Although not absolutely essential for constitutive secretion, Rab6 was previously reported to stimulate this MT-based transport of some PGCs from the Golgi/TGN and regulate their targeting to PM sites 36. Moreover, recent biochemical characterisation of PGCs has revealed a specific class of carrier that is positive for Rab6 45. Our data shows that Rab6 is required for the trafficking of HSV1 glycoproteins to the PM, and that glycoprotein-containing membranes on the way to the cell periphery contain Rab6. Interestingly, our studies on reporter proteins expressed by transient transfection revealed that the requirement for Rab6 in TGN-to-PM transport was variable, with transport of the tsVSV-G protein to the PM being severely attenuated in the absence of Rab6, but secretion of *Gaussia* luciferase being unaffected by its depletion. This is in agreement with other studies showing Rab6 is required for VSV-G transport to the PM 36,44, but is in contrast to Rab6 positive PGCs described in a recent publication which were shown to be distinct from VSV-G transporting carriers 45. This combination of results may point to Rab6 being critical in the transport of specific classes of proteins to the cell surface, particularly viral transmembrane proteins, but not luminal proteins.

There are two aspects to the utilization of Rab6 by HSV1 that have a consequence for our understanding of Rab6 function – firstly that Rab6 is necessary for glycoprotein transport to the PM, and secondly that HSV1 activates the Rab6-dependent transport pathway. In considering the requirement for Rab6 in post-Golgi trafficking, there are several points in the pathway at which its activity could be essential, including tubule formation, fission, transport on MTs and fusion at the PM. The non-processive motor myosin II has been implicated in efficient fission of Rab6 positive vesicles from the Golgi/TGN, and has been shown to be a component of a class of Rab6 positive PGCs 37,45. Moreover, myosin II has previously been identified as a potential binding partner of an HSV1 structural protein, while inhibition of myosin II activity was shown to reduce virus yield 46. Although this seemed a likely target to investigate, our studies here indicate that inhibition of myosin II activity, either via the drug blebbistatin, or siRNA knockdown of NMHCIIA and NMHCIIB, did not affect glycoprotein localization to the PM and resulted in only a twofold drop in virus production (a reduction that was specific to the NMHCIIB isoform) suggesting that any involvement of myosin II in virus envelope trafficking is limited. In agreement with these results, a very recent study on a class of PGCs shown to contain Rab6 and myosin II has also shown that myosin II is not required for the biogenesis of these PGCs 45, whereas the fission factor protein kinase D (PKD), which was also present in the PGCs, was required for their production 45,55,56. Moreover, as depletion or inhibition of PKD in HSV1 infected cells has been shown to reduce virus release 57, it seems likely that PKD, rather than myosin II, is involved in the fission of the Rab6 positive, HSV1 glycoprotein-containing membranes from the Golgi/TGN.

Our time-lapse analyses of infected cells indicate that virus infection may positively activate Rab6-dependent transport to the cell surface, with the number and length of Rab6 positive tubules leaving the Golgi/TGN being greatly enhanced, and long tubules frequently observed to leave the Golgi/TGN area, delivering a large amount of membrane to the cell surface. Such tubular PGCs have been described before in experiments using the tsVSV-G reporter 58,59, but not in the abundance observed here in HSV1 infected cells. These tubules undergo fission from the Golgi/TGN, and move to the PM on what appear to be specific tracks with rates appropriate for kinesin transport on MTs. The concentration of GFP-Rab6 and glycoproteins at the tips of infected cells is indicative of the presence of specific MT delivery routes to those regions of the cell periphery. Interestingly, although KIF5B, conventional kinesin heavy chain, has been shown to enhance Rab6 vesicle movement on microtubules 37, KIF5B depletion here had no effect on virus yield, suggesting there may be a potential redundancy among the kinesin motors that the virus can recruit for glycoprotein vesicle transport. Moreover, virus replication is known to continue in the absence of MTs in tissue culture monolayers at least, albeit to lower levels 60, suggesting that although MT-directed delivery to peripheral domains is required for optimal virus production, it is not absolutely essential. This has been shown to be the case for tsVSV-G, where nocodazole treatment of cells retarded but did not inhibit its delivery to the PM, which occurred at random sites rather than targeted regions of the PM 58. Nonetheless, the situation may be different in neurons where MTs are considered to be particularly important for the virus to transport its components down the relatively long axon to the cell termini for transfer to epithelial cells, although it is still a point of debate as to whether the virus is transported on axonal MTs as fully enveloped virions or as separate capsid and envelope entities 61–63. As neurons specifically express Rab6B 28, it would be of interest to determine if this isoform of Rab6 is important for HSV1 morphogenesis in neuronal cells.

In uninfected cells, the domains to where GFP-Rab6 positive vesicles are targeted have been shown to be enriched in the Rab6 binding partner ERC1 49, a cortical protein that plays an accessory role in linking distal MT ends to cortical platforms via CLASP proteins 64. It has been postulated that this interaction facilitates docking and/or fusion at the PM 36. It is of note then that the ERC1 effector was the only effector here that when depleted resulted in a drop in virus yield, reducing it by 10 fold, and causing a detectable reduction in gD levels at the cell surface, suggesting that ERC1 may facilitate optimal glycoprotein incorporation into the PM. Interestingly, a recent publication demonstrated the presence of sites on the PM which become enriched in glycoproteins early in HSV1 infection and which require an intact MT network to form 26. Moreover, ERC1 has been identified in proteomic screens for Dengue virus and hepatitis C virus interacting proteins 65,66, suggesting that ERC1 may be a common cellular factor utilized in the replication of enveloped viruses.

Rab6 is also involved in recruiting both the dynein–dynactin motor complex and the KIF20A kinesin like protein Rabkinesin-6 to the Golgi complex, but efficient depletion of the relevant Rab6 effectors – the dynactin components bicaudal-D1, D2 or dynactin 1 or KIF20A 42,47,48 – indicated that none of these factors are required in the HSV1 replication cycle. As Rab6 recruitment of dynactin by its interaction with bicaudal D and dynactin 1 is involved in Golgi-to-ER retrograde trafficking 35, these results indicate that there is likely to be no role for the retrograde trafficking of virus glycoproteins in the infected cell. Moreover, depletion of bicaudal-D2, but not -D1, caused a twofold increase in HSV1 virus yield, implying that disruption of the bicaudal-D2 interaction with Rab6 may allow more Rab6 to be recruited for the transport of HSV1-specfic PGCs. Interestingly, bicaudal-D1 and Rab6 have been implicated in the replication of the betaherpesvirus HCMV, by an interaction with its structural protein pp150 67,68. However, the fact that bicaudal-D1 does not appear to be involved in HSV1 replication points to these viruses using Rab6 by different mechanisms.

The results presented here on the role of Rab6 in HSV1 infection have allowed us to extend our recently published model of HSV1 envelopment where we described the wrapping of HSV1 capsids in membranes of the recycling endocytic network, and demonstrated that Rab5 is involved in the endocytic retrieval of virus glycoproteins from the cell surface 22. An important feature of this pathway is that virus envelope proteins must travel to the cell surface before reaching the final site of envelopment. Our results show that Rab6 is involved in transporting envelope proteins from the Golgi/TGN to the cell surface, prior to endocytosis to wrapping membranes. Hence, although endocytosis carries on normally in Rab6 depleted cells, the endocytosed membranes do not contain virus envelope or tegument proteins, and cannot function as wrapping membranes.

Given the profound effect of Rab6 depletion on HSV1 production, the molecular components of this pathway represent useful targets to understand HSV1 exploitation of the cellular secretory pathway and investigate new methods of interfering with virus replication. Moreover, HSV1 infection is likely to prove a useful and sensitive tool for investigation of the role of Rab6 in Golgi-to-PM transport, providing valuable insight not only into the cell biology of the virus–host relationship but also the activity of Rab6 itself.

## Materials and Methods

### Cells and viruses

HeLa cells and HaCaT cells were grown in DMEM supplemented with 10% foetal calf serum and antibiotics. nTERT keratinocyte cells 69 were grown in cells maintained in 3:1 DMEM:Ham's F12 supplemented with 10% foetal bovine serum, antibiotics, 10 ng/mL mouse epidermal growth factor (Serotec), 1 ng/mL cholera toxin (Sigma), 400 ng/mL hydrocortisone (Sigma), 5 mg/mL insulin (Sigma), 5 mg/ml transferrin (Sigma), 13 ng/ml liothyronine (Sigma) and 2 mm l-Glutamine (Invitrogen). HSV1 strains s17, sc16 and HFEM were routinely propagated and titrated in Vero cells in DMEM supplemented with 2% newborn calf serum and antibiotics. HSV1 (s17) expressing the glycoprotein gD as a GFP fusion protein has been described previously 22. All plaque assays were carried out in DMEM supplemented with 2% newborn calf serum and 1% human serum (Harlan Sera-Lab).

### Antibodies and reagents

Mouse antibodies to gD and gE were kindly provided by Helena Browne (Cambridge University) and David Johnson (Oregon Health and Science University) respectively. Rabbit antibody to VP22 (AGV031) has been described previously. Commercial antibodies used in this study were: giantin, NMHCIIA, NMHCIIB, bicaudal D1, KIF5B and dynactin 1 (Abcam); Rab1, Rab6A, bicaudal D2 and ICP0 (Santa Cruz Biotech); TGN46 (Serotec); Rab24 (BD Biosciences); alpha-tubulin and ERC1 (Sigma); KIF20A (Abnova); and VP5 (VirusSys). All Alexa fluor conjugated secondary antibodies, were obtained from Invitrogen. BFA, nocodazole, monensin and blebbistatin were obtained from Sigma and used at a concentration of 1 µg/mL, 1 µg/mL, 10 µg/mL and 50 µg/mL, respectively. Arabinofuranosyl cytidine (Ara C) was used at a concentration of 0.1 µg/mL.

### Plasmids and transfection

Plasmids expressing gD or gE under the control of the HCMV immediate-early promoter were kindly provided by Helena Browne (University of Cambridge). GFP-Rab6 expressing plasmid was kindly provided by Casper Hoogenraad (University of Utrecht). Plasmids expressing *Gaussia* luciferase (pCMV-GLuc-1) and GFP-tsVSVG were obtained from Nanolight (AZ, USA) and Addgene (plasmid 11912, Jennifer Lippincott-Schwartz) respectively. All plasmids were transfected into cells using lipofectamine 2000 (Invitrogen).

### siRNAs and transfection

Silencer Select siRNA duplexes (Table S1) were from Ambion and were transfected with Lipofectamine 2000 (Invitrogen) according to the manufacturer's instructions for reverse transfection. The final concentration of siRNA (2 duplexes per isoform) was 5 nm per isoform. The Silencer Select negative siRNA number 1 was used as a negative control.

### Reverse transcriptase-polymerase chain reaction

Poly A + mRNA was extracted from cells using an Oligotex Direct mRNA Mini Kit (Qiagen). Superscript III™ (Invitrogen) was used to make cDNA according to manufacturer's instructions, using random primers (Promega). Cycling was carried out in a Veriti 96 Well Thermal Cycler (Applied Biosystems) for 5 min at 25°C, 60 min at 50°C and 15 min at 70°C. All quantitative reverse-transcriptase polymerase chain reaction (qRT-PCR) assays were carried out using MESA GREEN qPCR MasterMix Plus for SYBR® Assay with dTTP (Eurogentec) using sense and antisense primers for Rab6A (F: acttggaggatcgaacaatcaggct; R: cagctgcagcagaatcacgga), Rab1A (F: tatgggacacagcaggccagg; R: acggaattccaagggaatcagc) and RPLP0 (F: actctgcattctcgcttcct; R: ggactcgtttgtacccgttg) as detailed in the manufacturer's instructions. qRT-PCR was carried out on an ABI7900HT qPCR machine (Applied Biosystems) with cycling parameters of 95°C for 15 min, then 95°C for 10 seconds, 60°C 15 seconds, 72°C for 15 seconds, repeated 40 times, 95°C for 15 seconds, and 60°C for 15 seconds. Results were analysed using Sequencing Detection System software v2.3 (Applied Biosystems).

### Semi-quantitative measurement of HSV genomic DNA

Viral DNA was purified from infected cells using a Qiagen Midi DNA kit. PCR was carried out on equal amounts of DNA using previously described primers that amplify a 300 bp fragment in the UL47 gene 32.

### Viability assays

Cell viability was assessed by flow cytometry using LIVE/DEAD Fixable Dead Cell Stain Kit (Invitrogen). Flow cytometry was carried out using a BD CyAn machine and analysed using Summit 4.3 software.

### SDS–PAGE and western blots

Protein samples were analysed by electrophoresis on polyacrylamide gels in Tris–glycine buffer followed by transfer to nitrocellulose membrane. Western blots were developed using an enhanced chemiluminescence kit (Pierce).

### Endo H treatment of virus glycoproteins

Cells were solublized in a buffer comprising 10 mm Tris/HCl (pH 7.5), 150 mm NaCl, 0.5 mm EDTA, 0.5% NP-40, digested with Endo H (NEB) according to the manufacturers instructions and analysed by SDS–PAGE and western blotting.

### Immunofluorescence

Cells for immunofluorescence were grown on coverslips and fixed with 4% paraformaldehyde in PBS for 20 min followed by permeabilization with 0.5% triton-X100 for 10 min. Endocytic structures were labelled by incubating cells with texas red conjugated transferrin (Invitrogen) at concentrations of 0.5 mg/mL in DMEM prior to fixation. Fixed cells were blocked by incubation for 20 min in PBS containing 10% newborn calf serum, and primary antibody added for 30 min in the same solution. Following extensive washing in PBS, the appropriate Alexa fluor conjugated secondary antibody (Invitrogen) was added in block solution and incubated for a further 20 min. The coverslips were then washed extensively in PBS and mounted in Vectashield containing DAPI (Vector Labs). Images were acquired using a 63× objective on a Zeiss LSM510 Meta confocal microscope, and processed using Adobe Photoshop software.

### Cell surface labelling

Culture supernatant of anti-gD monoclonal antibody LP14 was diluted 1 in 50 in DMEM without serum at 4°C, and added to cells on coverslips. The cells were left on ice for 30 min and washed twice before fixation for immunofluorescence.

### *Gaussia* luciferase assay

HeLa cells were transfected with plasmid pCMV-Gluc-1 and 24 h later the media was replaced and returned to 37°C. One hour later the extracellular media was collected and cell debris removed by centrifugation. Chemiluminescence was measured by addition of coelenterazine (Prolume Ltd.) at 1 µg/mL in PBS using an Autolumat Plus LB953 luminometer (Berthold Technologies). Relative light units (RLU) chemiluminescence/well was normalized to protein concentration/well, ascertained using the DC™ Protein Assay Kit (Bio-Rad Laboratories) as described in the manufacturer's instructions.

### VSV-G transport assay

HeLa cells grown on coverslips were transfected with siRNAs, and 2 days later transfected with plasmid pEGFP-VSV-G, that expresses the temperature sensitive form of the vesicular stomatitis virus G protein fused to EGFP. The cells were incubated at the non-permissive temperature of 39°C for 16 h, and then shifted to the permissive temperature of 32°C for 4 h, allowing the VSVG protein to be released from the ER into the secretory pathway. Samples were fixed and analysed as described above.

### Transmission electron microscopy

Cells for electron microscopy were fixed in 0.5% glutaraldehyde in 200 mm sodium cacodylate buffer for 30 min, washed in buffer and secondarily fixed in reduced 1% osmium tetroxide, 1.5% potassium ferricyanide for 60 min. The samples were washed in distilled water and stained overnight at 4°C in 0.5% magnesium uranyl acetate, washed in distilled water and dehydrated in graded ethanol. The samples were then embedded flat in the dish in Epon resin. Resin filled stubs were placed on embedded cell monolayers and polymerized. Ultrathin sections (typically 50–70 nm) were cut parallel to the dish and examined in a FEI Tecnai electron microscope with CCD camera image acquisition.

### Live cell imaging

Cells for live imaging were seeded onto glass bottom microwell dishes (MatTek Corporation). Sixteen hours after transfection with the GFP-Rab6 plasmid, the cells were infected with HSV1 (s17) at a multiplicity of 2, and left for 4 h. Live cell imaging was carried out by acquiring single slice images for times up to 12 h using a 63× objective on a Zeiss LSM510 Meta confocal microscope fitted with an environmental chamber. nih Image J software was used to construct videos from the original dataset.

## Supporting Information

Table S1 IDs of Ambion siRNA duplexes used to deplete human Rabs and Rab6 effectors from HeLa cells shown in Figures 1,6 and 7. Ambion siRNAs were obtained from Life Technologies.

Figure S1 Characterisation of Rab6 depletion in HeLa cells. A) HeLa cells transfected with Rab1 or Rab6 siRNAs were analysed 2 days after transfection by RT-PCR in comparison to negative siRNA transfected cells, using primers specific for the relevant Rab. B) HeLa cells transfected with negative, Rab1 or Rab6 siRNAs were analysed for cell viability 2 days after transfection. Graph denotes viability in relation to non-transfected cells (taken as 100%). C) HeLa cells transfected with negative or pooled Rab6 siRNAs were infected 2 days later with s17, sc16 or HFEM strains of HSV1, and virus release measured 16 h later. D) HeLa, nTERT or HaCAT cells were transfected with negative or pooled Rab6 siRNAs and infected 2 days later with HSV1 (s17) and virus release measured 16 h later. Error bars indicate standard error from three experiments. This figure relates to Figure 1 and confirms the efficiency of Rab6 depletion from HeLa cells, showing that Rab6 knockdown produces the same effect on virus production in two other relevant cell lines.

Figure S2 HeLa cells grown on coverslips were transfected with negative or pooled Rab6 siRNAs and infected 2 days later with HSV1 s17 expressing gD-GFP. Cells were fixed at the indicated time points and images acquired using a Zeiss LSM510 Meta confocal microscope. Scale bar = 20 µm. This figure relates to Figure 3 and shows a time course of gD localization in HeLa cells infected with gD-GFP expressing HSV1 in Rab6 depleted cells. These data confirm that gD failure to localize to the plasma membrane is consistent throughout infection.

Figure S3 HeLa cells grown on coverslips were transfected with negative or pooled Rab6 siRNAs and infected 2 days later with HSV1 s17 expressing GFP-VP22. Cells were fixed at the indicated time points and images acquired using a Zeiss LSM510 Meta confocal microscope. Scale bar = 20 µm. This figure relates to Figure 3 and shows a time course of VP22 localization in HeLa cells infected with HSV1 expressing GFP-tagged VP22, in cells previously transfected with Neg or Rab6 siRNAs.

Figure S4 Depletion of Rab6 has no effect on clathrin mediated endocytosis. HeLa cells transfected with Neg (upper panels) or Rab6 (lower panels) siRNAs were labelled 2 days later by incubating cells with texas red conjugated transferrin (0.5 µg/mL) for 30 min, followed by fixation. Endogenous Rab6 was labelled with Rab6 antibody (green), nuclei were stained with DAPI and images acquired using a Zeiss LSM510 Meta confocal microscope. This figure relates to Figure 4 and shows that while Rab6 depletion inhibits Golgi-to-PM transport, it has no effect on clathrin mediated endocytosis.

Video S1 HeLa cells expressing GFP-Rab6 by transient transfection were infected with HSV1 s17 at a multiplicity of 2, and imaged at 5 min intervals beginning 4 h after infection. Associated with Figure 8.

Video S2 Uninfected HeLa cells expressing GFP-Rab6 by transient transfection were imaged at 10 seconds intervals for a period of 30 min. Associated with Figure 9.

Video S3 HeLa cells expressing GFP-Rab6 by transient transfection were infected with HSV1 s17 at a multiplicity of 2, and imaged at 10 seconds intervals for a period of 30 min at 8 h after infection. Associated with Figure 9.

Video S4 HeLa cells expressing GFP-Rab6 by transient transfection were infected with HSV1 s17 at a multiplicity of 2, and imaged at 10 seconds intervals for a period of 30 min, 8 h after infection. Associated with Figure 9.

Video S5 HeLa cells expressing GFP-Rab6 by transient transfection were infected with HSV1 s17 at a multiplicity of 2, and imaged at 1 seconds intervals, 16 h after infection. Associated with Figure 9.
