# Supplementary material for: Rab6 Dependent Post-Golgi Trafficking of HSV1 Envelope Proteins to Sites of Virus Envelopment
Source: Traffic. 2013 Nov 18;15(2):157–78. doi: 10.1111/tra.12134 (PMC4345966; doi:10.1111/tra.12134)
Supplement: Table S1 — IDs of Ambion siRNA duplexes used to deplete human Rabs and Rab6 effectors from HeLa cells shown in Figures 1,6 and 7. Ambion siRNAs were obtained from Life Technologies. [file tra0015-0157-SD1.pdf]

Target	Duplex 1	Duplex 2	Target	Duplex 1	Duplex 2
RAB1A	s229381	s11658	RAB23	s28567	s28568
RAB1B	s117	s119	RAB24	s28803	s28804
RAB2A	s11660	s11661	RAB25	s32700	s32701
RAB2B	s39689	s39688	RAB26	s24590	s24591
RAB3A	s11666	s11667	RAB27A	s11693	s11694
RAB3B	s11669	s11671	RAB27B	s11696	s11697
RAB3C	s41884	s41885	RAB28	s17910	s17911
RAB3D	s18326	s18327	RAB30	s26136	s26137
RAB4A	s11675	s11676	RAB31	s21731	s21732
RAB4B	s28800	s28801	RAB32	s21618	s21619
RAB5A	s11678	s11679	RAB33A	s17907	s17908
RAB5B	s11681	s11682	RAB33B	s37937	s37938
RAB6A	s11685	s11686	RAB34	s38244	s38245
RAB6B	s28326	s28327	RAB35	s21707	s21708
RAB6C	s38489	s38490	RAB36	s18461	s18462
RAB7A	s15442	s15443	RAB37	s50262	s50263
RAB7B	s50333	s50334	RAB38	s24319	s24320
RAB8A	s8679	s8680	RAB39	s29359	s29360
RAB8B	s28633	s28634	RAB39B	s42008	s42009
RAB9A	s17916	s17917	RAB40A	s44480	s44481
RAB9B	s27691	s27692	RAB40B	s21588	s21589
RAB10	s21390	s21391	RAB40C	s33729	s33730
RAB11A	s16702	s16703	RAB41	s51268	s51269
RAB11B	s17647	s17648	RAB42	s41781	s41782
RAB12	s47368	s47369	RAB43	s50452	s50453
RAB13	s11690	s11691			
RAB14	s28311	s28312	NMHCIIA	s223	s224
RAB15	s51761	s51762	NMHCIIB	s9171	s9169
RAB17	s34608	s34609	BICD1	s1982	s1983
RAB18	s22704	s22703	BICD2	s23499	s23497
RAB19	s53588	s53589	KIF5B	s223974	s223975
RAB20	s31158	s31159	DCTN1	s52326	s3973
RAB21	s22823	s22824	ERC1	s22995	s22996
RAB22A	s32992	s32993	KIF20A	s19676	s19677

Table S1
