# Supplementary figures and images for: Rab6 Dependent Post-Golgi Trafficking of HSV1 Envelope Proteins to Sites of Virus Envelopment
Source: Traffic. 2013 Nov 18;15(2):157–78. doi: 10.1111/tra.12134 (PMC4345966; doi:10.1111/tra.12134)

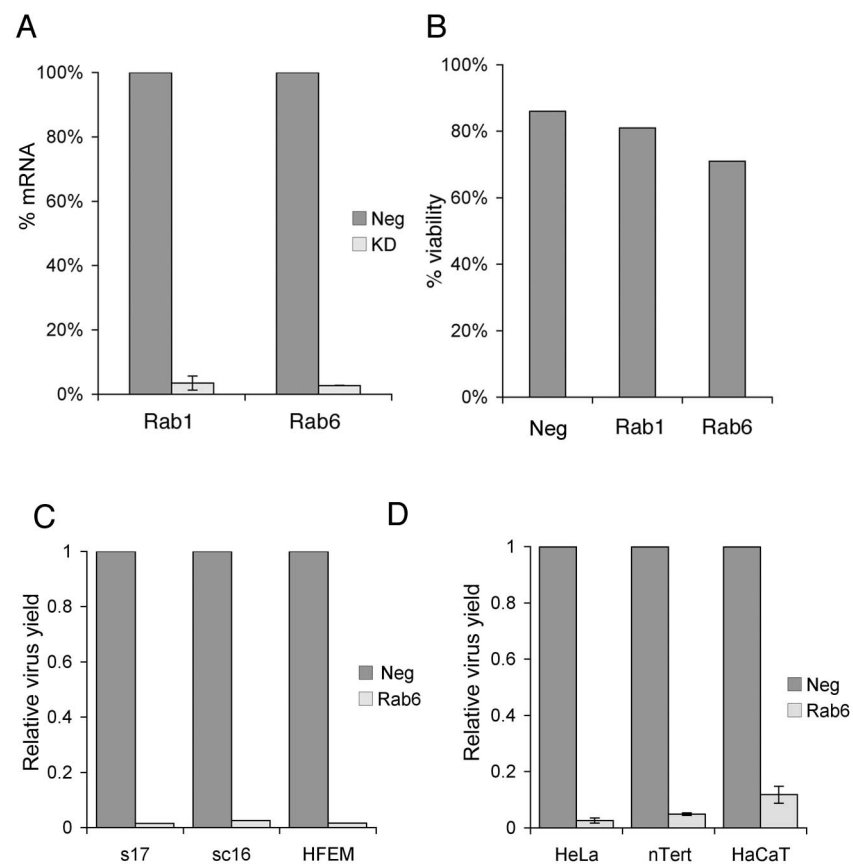

**Figure S1**

Supplement: Figure S1 — Characterisation of Rab6 depletion in HeLa cells. A) HeLa cells transfected with Rab1 or Rab6 siRNAs were analysed 2 days after transfection by RT-PCR in comparison to negative siRNA transfected cells, using primers specific for the relevant Rab. B) HeLa cells transfected with negative, Rab1 or Rab6 siRNAs were analysed for cell viability 2 days after transfection. Graph denotes viability in relation to non-transfected cells (taken as 100%). C) HeLa cells transfected with negative or pooled Rab6 siRNAs were infected 2 days later with s17, sc16 or HFEM strains of HSV1, and virus release measured 16 h later. D) HeLa, nTERT or HaCAT cells were transfected with negative or pooled Rab6 siRNAs and infected 2 days later with HSV1 (s17) and virus release measured 16 h later. Error bars indicate standard error from three experiments. This figure relates to Figure 1 and confirms the efficiency of Rab6 depletion from HeLa cells, showing that Rab6 knockdown produces the same effect on virus production in two other relevant cell lines. [file tra0015-0157-SD3.pdf]

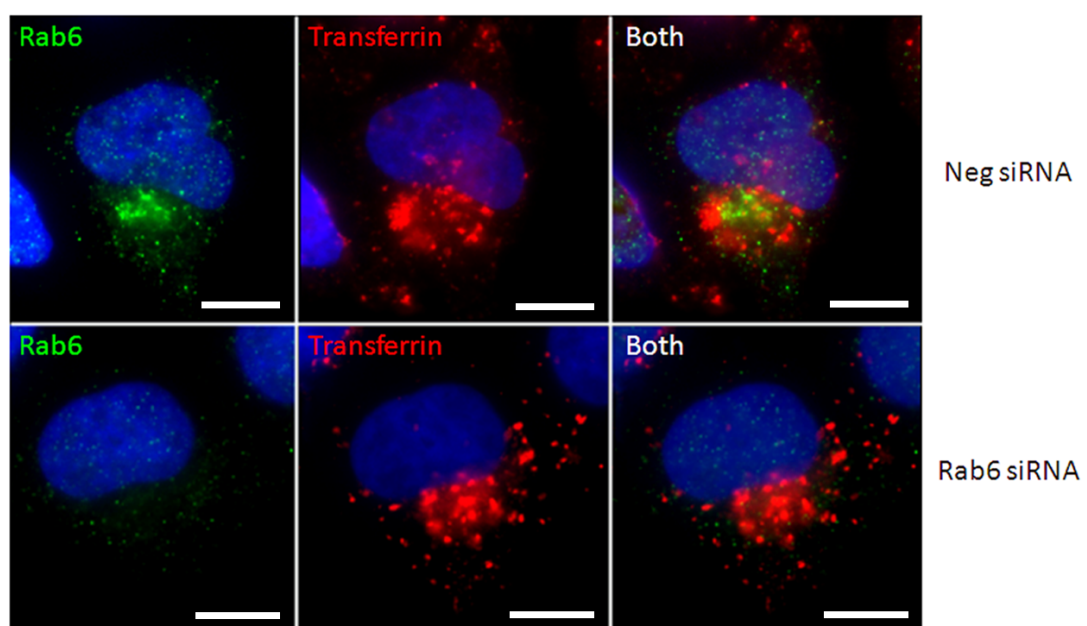

Figure S4

Supplement: Figure S4 — Depletion of Rab6 has no effect on clathrin mediated endocytosis. HeLa cells transfected with Neg (upper panels) or Rab6 (lower panels) siRNAs were labelled 2 days later by incubating cells with texas red conjugated transferrin (0.5 µg/mL) for 30 min, followed by fixation. Endogenous Rab6 was labelled with Rab6 antibody (green), nuclei were stained with DAPI and images acquired using a Zeiss LSM510 Meta confocal microscope. This figure relates to Figure 4 and shows that while Rab6 depletion inhibits Golgi-to-PM transport, it has no effect on clathrin mediated endocytosis. [file tra0015-0157-SD5.pdf]
